# Supplementary material for: Successfully Recruiting Adults with a Low Socioeconomic Position into Community-Based Lifestyle Programs: A Qualitative Study on Expert Opinions
Source: Int J Environ Res Public Health. 2020 Apr 16;17(8):2764. doi: 10.3390/ijerph17082764 (PMC7215437; doi:10.3390/ijerph17082764)
Supplement: Supplementary file 1 [file ijerph-17-02764-s001.pdf]

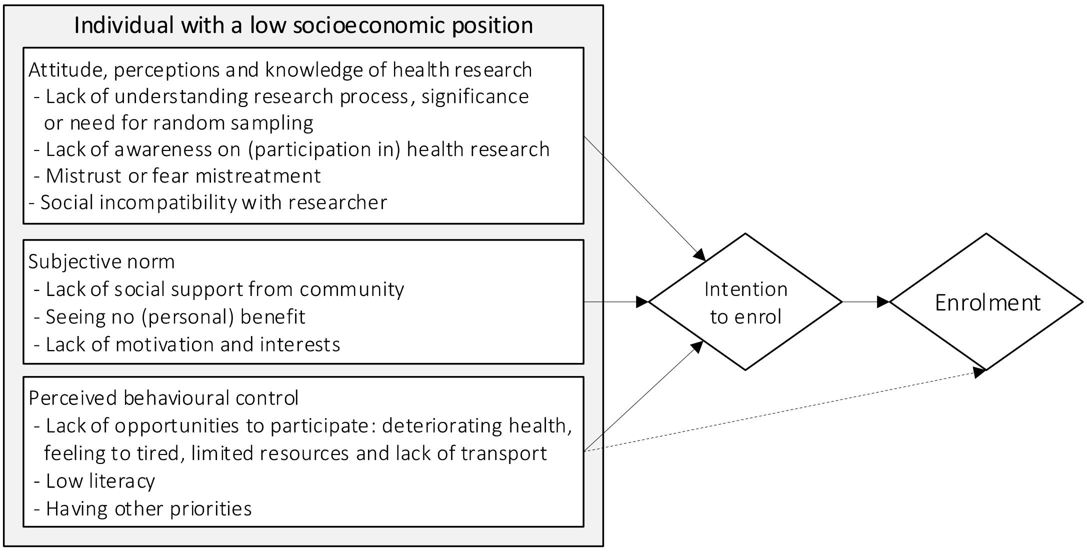


**Supplementary Figure 1**. Conceptual model as basis for the initial codebook: Predefined themes based on the Theory of planned behaviour and initial coding derived from literature.
